# The MEC-2E isoform with a large C-terminal completely rescues the touch sensation defect of *C. elegans*

**DOI:** 10.1038/s41598-025-10711-w

**Published:** 2025-07-22

**Authors:** Tália Magdolna Keszthelyi, Regina Légrádi, Dóra Pálya, Tímea Köles, Ágnes Regős, Dóra Karancsiné Menyhárd, Kálmán Tory

**Affiliations:** 1 MTA-SE Lendület Nephrogenetic Research Group, Budapest, Hungary; 2 Pediatric Center, MTA Center of Excellence, Semmelweis University, Budapest, Hungary; 3 HUN-REN-ELTE Protein Modeling Research Group, ELTE Eötvös Loránd University, Budapest, Hungary; 4 Medicinal Chemistry Research Group, HUN-REN Research Centre for Natural Sciences, Budapest, Hungary

**Keywords:** Functional transcript, Mechanosensation, Chemotaxis, Mec-2, Alternative splicing, Genetics

## Abstract

Human podocin and *C. elegans* MEC-2 belong to the stomatin protein superfamily. They share 49% identity and 91% similarity both in the evolutionary conserved PHB domain (123-284 aa) and in the oligomerization region (273-351 aa). Amino acid substitutions in these conserved regions can modify the podocin oligomerization and thus the pathogenicity of *trans-*associated *NPHS2* variants, known as interallelic interactions. The MEC-2A isoform was formerly considered to be the functional isoform and used to evaluate the effect of pathogenic podocin variants. The *mec-2* mutant worms are mechanosensation deficient, and, as recently described, also chemosensation deficient. To study the interallelic interactions of podocin in vivo, we aimed to rescue the phenotype of the *mec-2* mutant worm by reexpressing podocin (383 aa). However, we did not detect any chemotaxis defects in *mec-2(u37)* null mutants nor in *mec-2(e75)* missense mutants. No mechanosensation rescue was achieved by MEC-2A, but with a 17,5 kb genomic region and the MEC-2E isoform (1239 aa) with a large C-terminal. Truncating the last third of the large C-terminal abolished its rescue effect. In conclusion, the function of MEC-2 in mechanosensation requires a large C-terminal encoded by the MEC-2E isoform. Accordingly, human podocin cannot rescue the phenotype of *mec-2* mutants.

**Supplementary Information:**

The online version contains supplementary material available at 10.1038/s41598-025-10711-w.

## Introduction

The podocin encoding *NPHS2* is the most frequently mutated gene in steroid-resistant nephrotic syndrome^1^. While *NPHS2* variants typically cause autosomal recessive nephrotic syndrome, specific *trans*-associations of its variants may inverse the pathogenicity^2^. Accordingly, we formerly found the R229Q variant to be subject of a dominant negative effect when associated to specific 3’ missense variants, rendering this benign variant pathogenic^2^. On the other hand, the internalization of the F344fs podocin was prevented by the membrane-localized R238S podocin, raising the possibility of interallelic complementation between two pathogenic variants^3^.

It is crucial for the clinical practice to differentiate pathogenic and benign *NPHS2* associations to evaluate the necessity of immunosuppression or the possibility of recurrence after renal transplantation. However, interallelic interactions are not even taken into account while interpreting the pathogenicity of sequence variants^4^. To help their evaluation, we aimed to establish a *C. elegans* model, allowing the generation and crossing of numerous missense mutants and studying the pathogenicity of their *trans*-associations. The *C. elegans* homologue of human *NPHS2* is *mec-2*^1,5^. Similarly to podocin, MEC-2 has a highly specialized function and is expressed in six touch receptor neurons^5^ and as described recently, in some olfactory^6,7^ and thermosensory^8,9^ neurons. The *mec-2* null mutant worms are gentle touch insensitive^5^. Podocin and MEC-2 are members of the SPFH (stomatin, prohibitin, flotillin, HflK/C) / Band-7 protein superfamily and share a high degree of similarity in their conserved SPFH (or PHB) domain^1,5,10^. SPFH/PHB domain proteins are generally integral membrane proteins with cholesterol-binding motifs that tether them to lipid rafts. In mammals they regulate various mechanosensitive channels and transport membrane proteins, like glucose transporter GLUT-1 (stomatin), anion exchanger AE-1 (stomatin) or the PIEZO2 channels (mouse STOML3)^11–15^. *C. elegans* MEC-4 and MEC-10 proteins form a sensory mechanotransduction channel, the activity of which is regulated by MEC-2^16,17^. MEC-2 is also responsible for the cholesterol binding of the protein complex that stabilizes the localization of the channel in the lipid rafts^18^. Human podocin is associated to TRPC6, a Ca^2+^ channel in the slit diaphragm and regulates its activity in a cholesterol dependent manner^19,20^. The molecular mechanism of the regulation is not elucidated yet in details, it may be implemented by changing the local lipid environment around the channel^21^. Characteristics of podocin and MEC-2 are compared in Table S2.

The MEC-2 protein has 17 predicted isoforms produced from a single gene via alternate splicing (https://www.ensembl.org/Caenorhabditis_elegans/Gene/Summary?db=core;g=WBGene00003166;r=X:5567754-5590830), with MEC-2A considered to be the functional one^5,22^. All 17 share a central part with the SPFH/PHB domain. Secondary to alternative splicing, catalysed by the MEC-8 protein that removes the ninth intron, two major MEC-2 isoform types can be distinguished: short and long ones. The *mec-8* null mutant strains express only the short isoforms (MEC-2B, C, K, L) and are touch insensitive^22^.

To establish an in vivo model of interallelic interactions we aimed to rescue the touch-insensitive phenotype with human podocin. Here we show that neither human podocin nor MEC-2 A can rescue the touch sensation deficiency, but only the MEC-2E with a long C-terminal. We also show that no chemotaxis deficiency can be detected in *mec-2* mutants and the integrity of the C-terminal is essential for gentle touch sensation.

## Results

### Neither podocin, nor MEC-2A rescue touch insensitivity

**Fig. 1:**
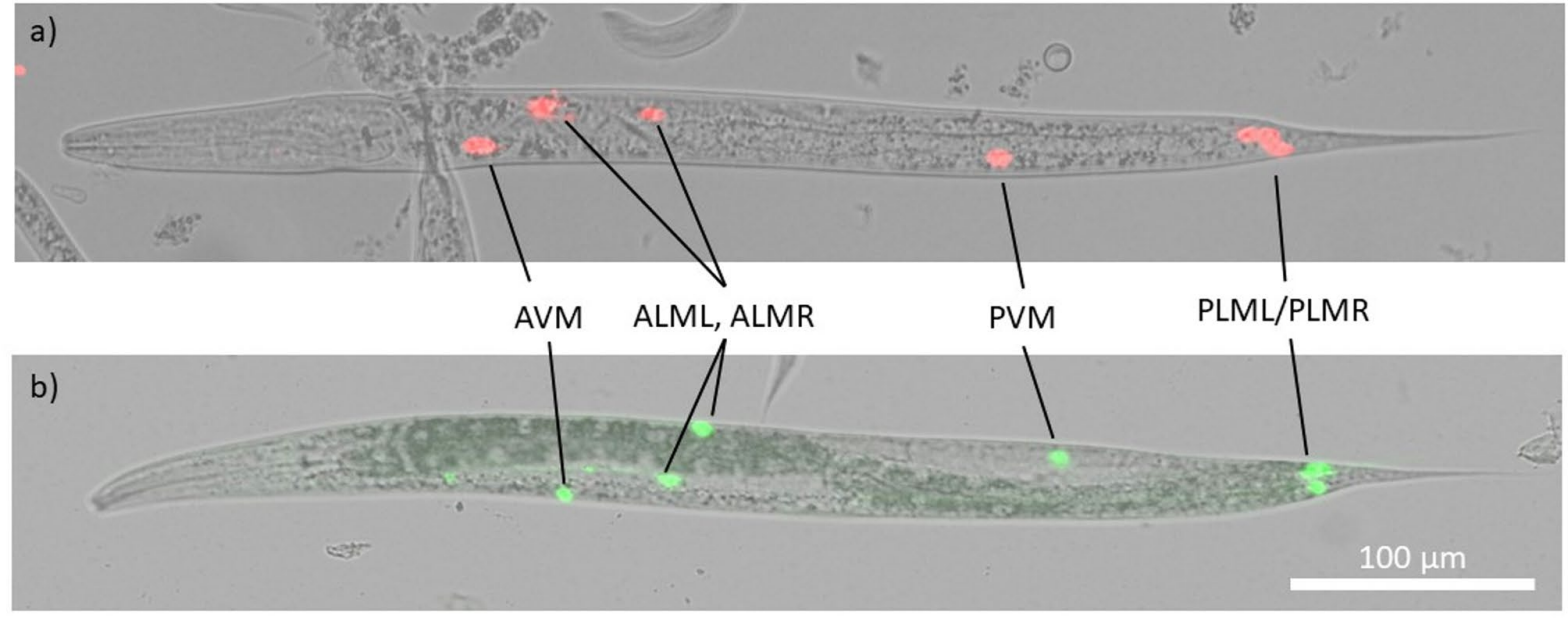
Validation of the *mec-2* promoter with either Pmec-2::mCherry (**a**) or Pmec-2::GFP (**b**). The expression pattern of the fluorescent proteins corresponds to the localization of the six touch-sensitive neurons (AVM, ALML, ALMR, PVM, PLML, PLMR) in both lines, indicating the specificity and efficacy of the promoter. No expression was seen in the olfactory and the thermosensory neurons.

After validating the efficiency of the MEC-2A promoter (*Pmec-2*) (Fig. 1) we expressed human podocin-mCherry and MEC-2A::GFP in the *mec-2(u37)* strain. A proper expression of the podocin-mCherry was only achieved when artificial introns were inserted in the cDNA construct (Table S1). Nevertheless, no rescue was achieved with any of the two constructs. Cleavage of the fluorescent tags by insertion of the T2A sequence did not improve the rescue effect either^23^. To ensure a proper level of expression, we combined MosSCI technique with microparticle bombardment^24^. Single copy insertion was thus achieved with both constructs, though the efficiency of MosSCI seemed lower with microparticle bombardment than the reported efficiency with microinjection^24–26^. Despite all these efforts, not only the human podocin, but also the MEC-2A failed to rescue the touch insensitivity. We thus concluded that the MEC-2A isoform is not the functional one.

### Identification of the functional MEC-2 isoform

**Fig. 2:**
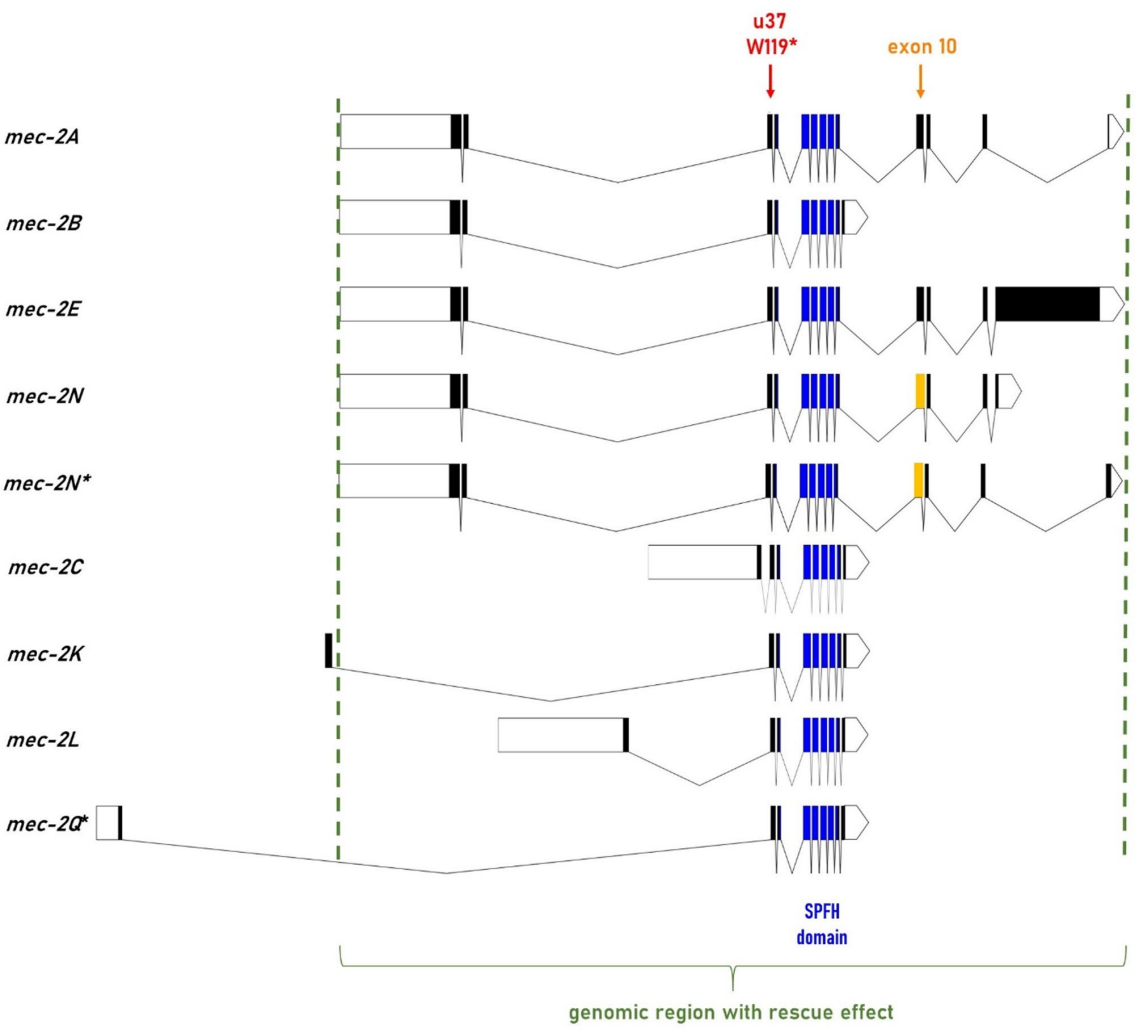
Splice isoforms of *mec-2.* Out of the 17 *mec-2* splice variants listed in ensembl.org, we found seven and two additional ones (*) to be expressed at the RNA level. The *u37* W119* variant (red arrow) and the SPFH/PHB domain (in blue) are present in all isoforms. The borders of the 17.5 kb genomic sequence with a rescue effect are indicated by green dashed lines. Exon 10 of the long isoforms differs in its size as indicated by different colors: it is 13 bp longer in the *mec-2N* and mec-2N* splice variants (in orange) than in the *mec-2A* and *mec-2E* (in black), shortening the C-terminal of N isoform compared to E, and lengthening that of the N* compared to A.

We next aimed to identify the functional MEC-2 isoform. We generated a giant expression vector, containing the complete ~ 17,5 kb genomic sequence of the MEC-2E and MEC-2A isoforms under the same *Pmec-2* promoter (Fig. 2), but not the first exon of some other splice variants (MEC-2Q, M, K, H, D) listed by ensembl.org. This vector rescued the touch sensation defect, even without genomic integration. Out of the 17 MEC-2 splice isoforms listed in ensembl.org, we could amplify seven from total RNA of wild type worms (Fig. S1). The remaining ten splice variants for which no PCR product was obtained were considered either non-existent or expressed at a low level that precluded their functionality. We found two additional isoforms: Q* with a 5’ region identical to the Q isoform, but encoding a short C-terminal, and N* that differs from the N isoform only in the last exon (Fig. 2, Fig. S1).

**Fig. 3:**
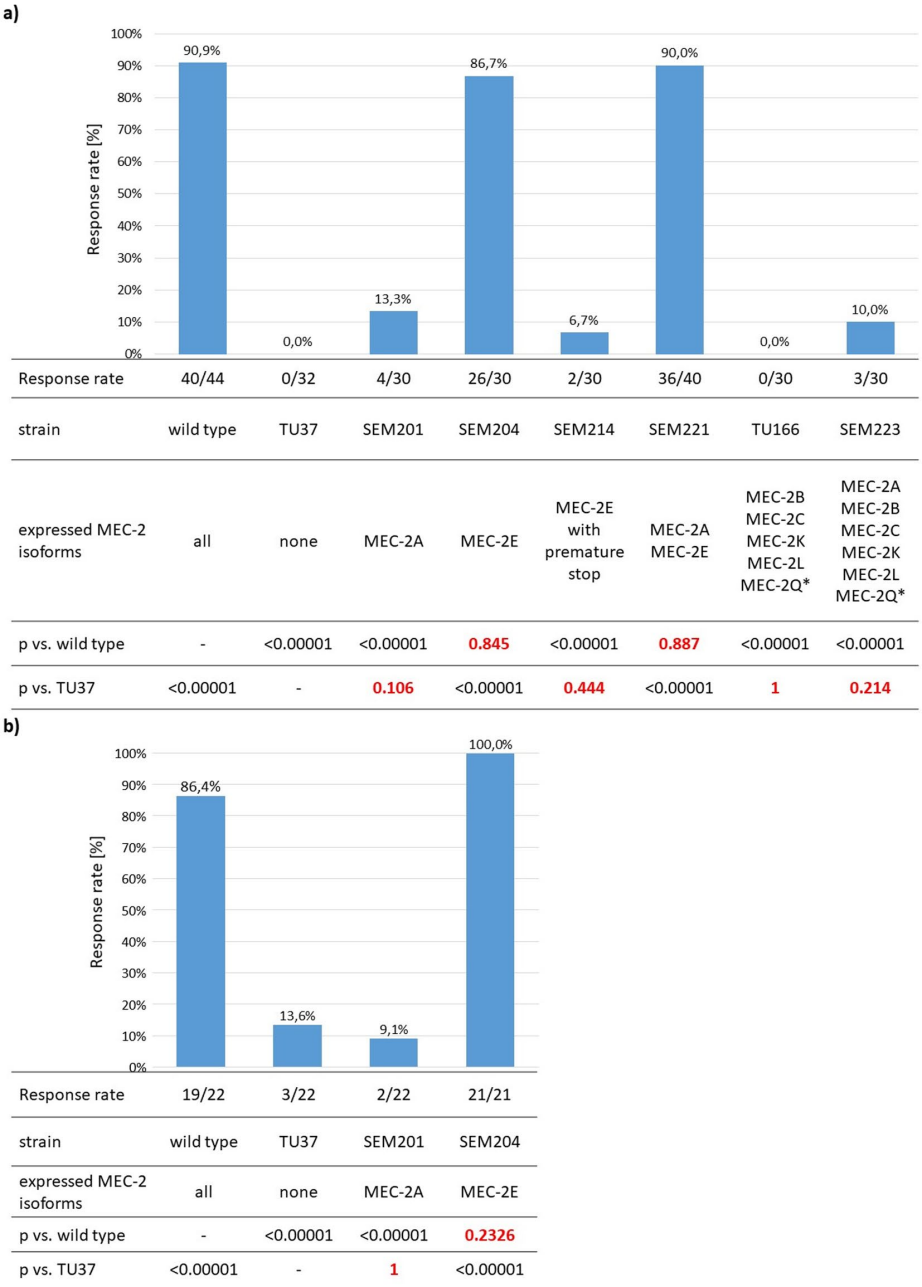
Response rate to gentle touch stimuli in function of the expressed MEC-2 isoform. Touch stimuli were exerted with either cat’s whisker (**a**) or “eyebrow on a toothpick” (**b**). The *mec-2* nonsense mutant (TU37) and the *mec-8* nonsense mutant (TU166) animals are touch insensitive. While MEC-2A had no rescue effect, MEC-2E completely rescued the gentle touch deficiency. Truncation of the C-terminal in the MEC-2E isoform (SEM214) abolished its rescue effect. MEC-2A isoform had no additional effect when coexpressed with either MEC-2E (SEM221) or in TU166 (SEM223).

Out of the nine isoforms, the five encoding a short C-terminal (MEC-2B, MEC-2C, MEC-2K, MEC-2L and MEC-2Q*) do not require MEC-8 (Fig. 2)^22^. The mechanosensation deficiency of *mec-8(u314)* worms therefore reflects the lack of their functional role. Along this line, even the reexpression of MEC-2A in the *mec-8(u314)* worms did not rescue the phenotype, indicating the lack of their combined rescue effect (Fig. 3). Three transcripts remained as the potential functional transcript (MEC-2E, MEC-2N or MEC-2N*). MEC-2N and N* only differ from MEC-2A in a short C-terminal sequence of 58 and 105 residues, respectively, but the MEC-2E contains a large C-terminal (764 residues), suggesting its potential role in mechanosensation. Indeed, MEC-2E rescued completely the touch sensation deficiency as validated in blinded experiments either by cat’s whisker or human eyebrow by two-two researchers after random integration of *mec-2E* coding sequence. In accordance with its functional role, we found by qPCR the *mec-2E* to be expressed at a similar level to *mec-2A* in wild type worms. Since Liang et al.^6^ recently proposed the combined rescue effect of MEC-2A and MEC-2E, we investigated their combined effect, but found no additional benefit from the coexpression of MEC-2A (Fig. 3). To ascertain the pivotal role of the C-terminal of MEC-2E, we truncated the last third of the C-terminal by generating a premature stop codon (c. 3076-77CC > TG; p.P1026*, *mec-2(seu1026)*), which failed to rescue the touch insensitivity. We thus conclude that the long C-terminal of MEC-2E is indispensable for mechanosensation, and this isoform alone can completely rescue the phenotype.

### Structural model of MEC-2E C-terminal segment

To explore the significance of this region we predicted with AlphaFold3^27^ the monomeric and multimeric structure of MEC-2E. In the predicted structure of the monomer (Fig. S2), residues 1027–1239 form a long, mostly unstructured tail, which also hosts 3 helical segments. However, in the predicted structure of a tetramer, the proximity of the C-terminal tails enhances helicalization and these ordered segments proceed to form a helical “buttress” supporting the aligned PHB domains via extended inter-chain contacts. This presents a possible scenario where the extra-long C-terminal tail of MEC-2E might provide support or readjustment of the homo-multimeric associations. This predicted model resembles the recently determined cryo-EM structures of SPFH proteins that self-associate into of helical pore structures, such as flotillin1/2^28^, prohibitin1/2^29^ and HflK/C^30,31^ proteins. Since MEC-2 is a regulator of sensory ion channels^16,32 ^it might not form a pore-like macrostructure like flotillin, prohibitin or HflK/C. Nevertheless, these simple structural considerations together with the in vivo results suggest that the C-terminal segment most likely contributes to the stabilization of the homomultimeric form, supporting the emergence of more robust assemblies.

### The role of *mec-2*/MEC-2 in chemotaxis

**Fig. 4:**
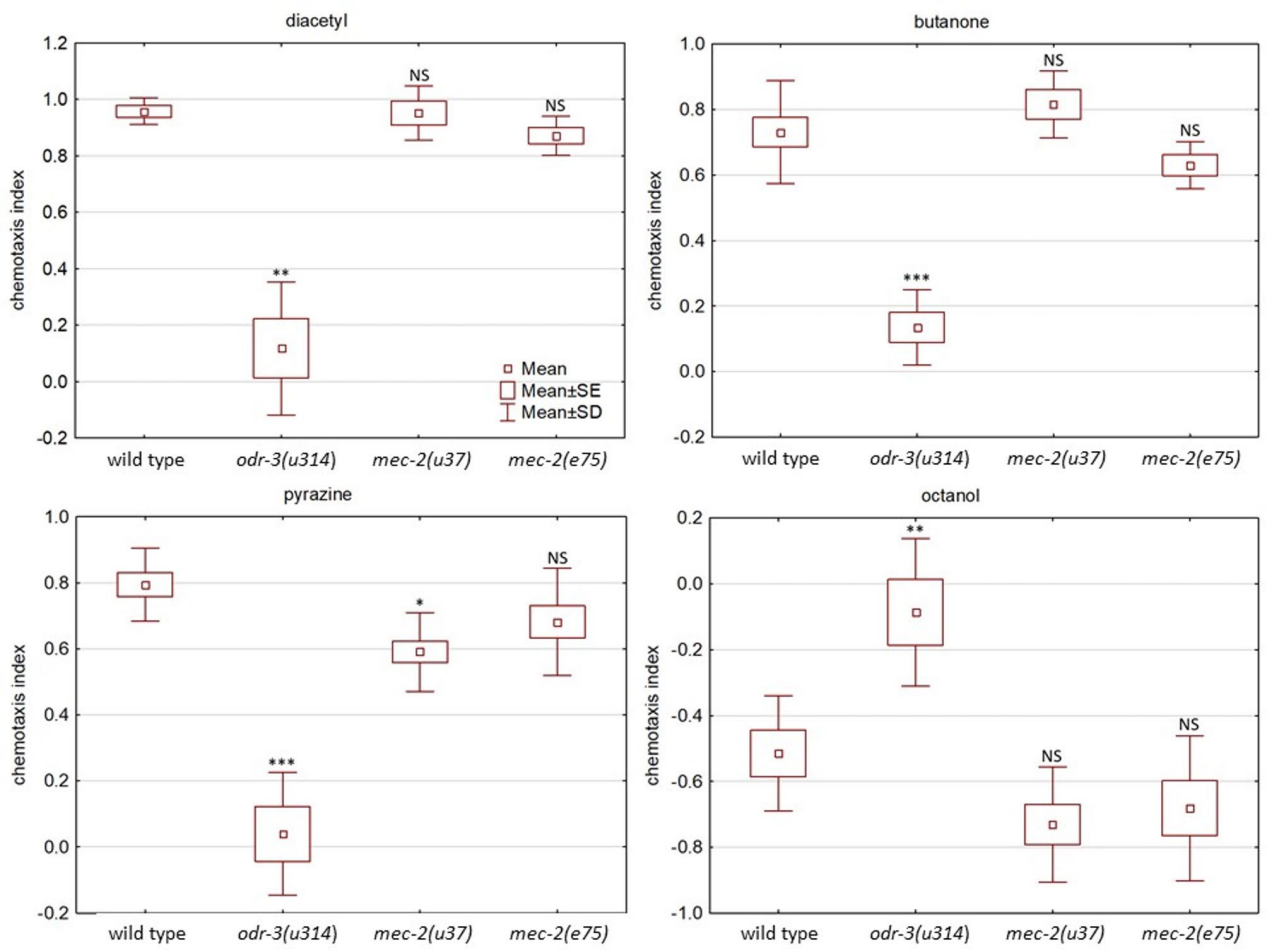
The chemotaxis behavior of *mec-2* mutant worms. The chemotactic behavior of the *mec-2(u37)* and *mec-2(e75)* worms was similar to the wild type, and markedly different from that of the chemotaxis deficient *odr-3(n2150)* animals. (NS: not significant, *P < 0.05, **P < 0.01, ***P < 0.001 vs. the wild type)

The role of MEC-2 in chemotaxis was recently suggested by Liang et al.^6 ^as its short MEC-2B isoform is expressed in olfactory neurons. This prompted us to investigate the rescue effect of podocin on chemotaxis defect, since the MEC-2B isoform (392 aa) largely overlaps with human podocin with 39% identity and 78% similarity, both containing the highly conserved SPFH/PHB domain and oligomerization region but not the non-conserved C-terminal. We intended to make the chemotaxis assay developed by Margie et al.^33^ more specific by counting the worms only in a defined radius around the odorant drop, instead of the quarter circle of the Petri dish (Fig. S3). An experiment was considered conclusive if 50–250 worms moved in the test areas in the original setup, or 20–150 worms in the modified one. Experiments with the lethargic *mec-2(e75)* strain were often inconclusive with octanol and in the control experiment (with ethanol) and needed to be repeated several times for reliable assessment (octanol: *mec-2(e75)*: *n* = 13/25 (inconclusive/all), wt: *n* = 0/10, *p* = 0.0073; control: *mec-2(e75): n* = 16/25, wt: *n* = 2/10, *p* = 0.042). Nevertheless, the chemotaxis indexes obtained in conclusive tests of both *mec-2* mutant strains to diacetyl, butanone and octanol were comparable to the wild type (Fig. 4). A biologically negligible reduction in the chemotaxis response to pyrazine was found in the *mec-2(u37)* strain, which is not comparable to that of the *odr-3(n2150)* strain with a markedly impaired chemotaxis to all four odorants (Fig. 4). Finally, counting the worms in the quarter circles according to Margie et al.^33^ did not affect the chemotaxis response rates. We therefore found no significant chemotaxis defect neither in the nonsense mutant (*mec-2(u37)*), nor in the missense mutant (*mec-2(e75)*) strain (Fig. 4). According to Liang et al.^7^ we also performed ‘smell on a stick’ test with octanol on both *mec-2(e75)* and *mec-2(u37)* animals. Though the *mec-2(e75)* worms were too lethargic to evaluate their response, the *mec-2(u37)* worms reacted similarly to the wild type and differently from the *odr-3(n2150)* strain. The lack of a chemotaxis defect in the *mec-2* mutant strains provided no possibility to examine the rescue effect of human podocin.

## Discussion

Interallelic interactions can reshape the pathogenicity of sequence variants in specific associations. This mechanism makes challenging the pathogenicity assessment of sequence variants^34^ which may require functional experiments. For this, it is essential to identify the canonical isoform, that is expected to exert all gene-related functions. Having rarely the possibility to study the function of the encoded protein, the canonical transcript is generally selected based on one of the following criteria^35^: greatest length^36^, highest expression level^37,38^, widest expression pattern or highest evolutionary conservation^39^. Genomic databases (Uniprot^40^, Ensembl^41^, NCBI RefSeq^42^) combine these criteria with different weighting. Expression level- and conservation-based isoform selection often contradict the sequence length-based ones^35,39,43,44^. Li et al. found a conservation- and expression level-based approach to be the most efficient in the selection of the functional isoform in 3427 multi-isoform mouse genes^35^. Assuming a ‘one for all‘ function may be overly simplistic for some genes, as it is for several genes like *WT1*, the function of which necessitate multiple transcripts^45–51^ or *BBS8*, with a specific splice variant necessary for its function in the retina^52,53 ^etc. Nevertheless, expression of a single mRNA can typically rescue the phenotype of KO animal models, suggesting that canonical transcripts do exist for the majority of the genes^54–59^.

*C. elegans* MEC-2 was formerly used to model the functional consequences of podocin amino acid changes^21,60^. Indeed, MEC-2 and podocin share 49% identity and 91% similarity in both the SPFH/PHB domain (podocin 123–284 aa) and the oligomerization region (podocin 273–351 aa). Over the conserved 250 aa (podocin 98–347 aa) there is 48% identity and 90% similarity. The MEC-2A isoform, composed of 481 aa, was considered to be the functional one^5^. However, until recently no functional studies had been done to support it. We could not rescue the touch sensation deficiency by reexpressing MEC-2A in *mec-2(u37)* worms. In accordance with our results, Liang et al. found no rescue after forced expression of the MEC-2A isoform either^6^.

We first achieved successful rescue of the touch insensitive phenotype with a 17,5 kb large genomic region, encoding seven out of the nine isoforms that we found expressed at a detectable level. Out of the four isoforms (MEC-2A, MEC-2E, MEC-2N and MEC-2N*) that are encoded by the 17,5 kb large genomic region and absent in the *mec-8(u314)* null mutant worms, we found the MEC-2E isoform to completely rescue the phenotype. This isoform (1239 aa) contains a large C-terminal that is twice the size of the entire MEC-2A. Generating a premature stop (p.P1026*) within the 3’ third of C-terminal abolished its rescue effect, further confirming its importance.

Intriguingly, Liang et al. found the touch sensation deficiency to be rescued only by the combined expression of the MEC-2A and MEC-2E isoforms^6^. The MEC-2E isoform alone had only a partial effect in their experiments. While Liang et al. used CRISPR/Cas9-based forced expression in wild type worms to ensure the selective expression of certain isoforms^6^, we overexpressed the MEC-2E isoform after random integration in the null (*mec-2(u37)*) mutant. As MEC-2E completely rescued the mechanosensation deficiency in our experiments, the combined expression of MEC-2A had no additional effect. The assumption that the combined expression of two different isoforms is required for the proper function of a gene in an AR disorder seems unlikely. This would mean that either of two pathogenic variants, each affecting only one of the two isoforms, would be pathogenic in the homozygous state, but not when *trans*-associated to each other, in the compound heterozygous state. However, to our knowledge, no incomplete penetrance has ever been reported in an AR disorder resulting from this mechanism. Along this line, a single mRNA can typically rescue the phenotype of KO animals, suggesting a single canonical transcript^54–59^.

Liang et al. found the *mec-2(e75)* strain to be olfactory deficient^6,7^. Chemotaxis deficiency was described formerly in the *mec-2(e75)* missense mutant animals^6,7,61^. This could be rescued by the short MEC-2B isoform lacking the C-terminal with a length of 392 aa and containing the entire SPFH/PHB domain^6^. This prompted us to test the rescue with podocin of similar size (383 aa) and high similarity. However, we found no chemotaxis defect by four different odorants in the *mec-2(e75)*, or in the *mec-2(u37)* mutant strains. The *mec-2(e75)* strain is notoriously lethargic, especially in the presence of bacteria^6,62^. This lethargic behavior may bias the chemotactic tests, making them false positive, especially in the presence of residual bacteria after washing. For this reason, we had to repeat the test several times to achieve a sufficient number of moving *mec-2(e75)* worms. The *mec-2(u37)* worms showed a mild chemotaxis defect to only one of the four odorants, but the *mec-2(e75)* mutant worms perfectly reacted to all four. Based on these experiments, the lethargic *mec-2(e75)* strain may not be an appropriate model in behavioral tests.

In conclusion, we found the MEC-2E isoform to completely rescue the phenotype and consider it to be the functional transcript. This isoform is neither expressed more widely^6 ^nor is it more conserved than MEC-2A. Only its greater length could have indicated its canonical role. The contradictory results of several aspects well reflect the necessity of in vivo functional tests in the canonical isoform selection. The large C-terminal of the MEC-2E explains why podocin, with no homologous segment, cannot rescue the touch sensation defect. Unfortunately, we found no chemosensation defect associated to the deficiency of the short isoforms. We therefore could not use the *mec-2* deficient *C. elegans* to study the interallelic interactions of podocin.

## Materials and methods

### *C. elegans* strains*

All strains were bred at 20 °C on NGM plates with 0.1% streptomycin and fed with streptomycin resistant OP50 bacterium (HB101 strain, CGC).

N2 (Bristol) as wild type (purchased from CGC).

TU166 *mec-8(u314) I.* (purchased from CGC)

CB75 *mec-2(e75) X.* and TU37 *(mec-2(u37) X*. were kindly provided by the laboratory of M. Chalfie, (Department of Developmental Biology, Columbia University, New York)

CX2205 *odr-3(n2150) V.* was kindly provided by the research group of Cs. Sőti (Department of Molecular Biology, Semmelweis University, Budapest).

EG6701 *ttTi4348 I; unc-119(ed3) III; oxEx1580.* (purchased from CGC)

EG6699 *ttTi5605 II; unc-119(ed3) III; oxEx1578.* (purchased from CGC)

SEM191 *ttTi4348 I; unc-119(ed3) III; mec-2(u37) X.* (generated by crossing of EG6701 and TU37)

SEM192 *ttTi5605 II; unc-119(ed3) III; mec-2(u37) X.* (generated by crossing of EG6699 and TU37)

### Transformant strains were generated by the combination of MosSCI technique and microparticle bombardment

SEM201 *unc-119(ed3) III; mec-2(u37) X.; seuIs01[Pmec-2::mec-2 A cDNA; cbr-unc-119(+)]*

SEM204 *unc-119(ed3) III; mec-2(u37) X.; seuIs02[Pmec-2::mec-2E cDNA; cbr-unc-119(+)]*

SEM215 *ttTi5605 II; unc-119(ed3) III;; mec-2(u37) X.; seuEx03[mec-2E gDNA; cbr-unc-119(+)]*

SEM214 *ttTi4348 I; unc-119(ed3) III; mec-2(u37) X.; seuEx04[Pmec-2::mec-2E(seu1026); cbr-unc-119(+)]*

### Double mutants were generated by crossing

SEM221 *unc-119(ed3) III; mec-2(u37) X.; seuIs01[Pmec-2::mec-2 A cDNA; cbr-unc-119(+)]; seuIs02[Pmec-2::mec-2E cDNA; cbr-unc-119(+)]*

SEM223 *mec-8(u314) I.; unc-119(ed3) III; mec-2(u37) X.; seuIs01[Pmec-2::mec-2 A cDNA; cbr-unc-119(+)]*

### Vector construction

The *mec-2* promoter sequence containing vector was kindly provided by the laboratory of M. Chalfie. The promoter function was validated by studying GFP or mCherry expression with Modular Stereo Microscope for Fluorescent Imaging (Leica MZ10F).

NEBuilder DNA assembly kit and Q5 Site Directed Mutagenesis Kit (New England Biolabs) were used to generate specific expression vectors. Phusion High-Fidelity PCR Master Mix or Q5 Hot Start High-Fidelity 2X Master Mix was used for the PCR reactions. Vector sequences were validated after editing by Sanger sequencing, with particular regard to the specific coding and promoter regions.

Vectors for the MosSCI (Mos1-mediated Single Copy Insertion)^24^ technique (pCFJ350, pCFJ352 and pCFJ601) were obtained from Addgene.

The *mec-2A* and *mec-2E* coding sequences were amplified from cDNA, the 17,5 kb genomic sequence from gDNA of N2 worms. The *NPHS2* coding sequence was codon optimized (https://worm.mpi-cbg.de/codons/cgi-bin/optimize.py)^63^ and synthesized by IDT. The second codon optimization resulted in appropriate podocin expression (Table S1), as controlled by C-terminal mCherry or GFP. To increase the expression efficiency, three artificial introns were inserted in the *mec-2A*, *mec-2E*,* mec-2E(seu1026)* and human *NPHS2* coding sequences. The 17,5 kb genomic sequence (X: g.5579230.5596676; 17447 bp) encodes the majority of the isoforms with the exceptions of MEC-2K, MEC-2Q* (Fig. 2). Primer sequences are available upon request.

### Transformation, selection and scrutiny of the worms

Worms were transformed by microparticle bombardment (Bio-Rad PDS-1000 | He™ and Hepta™ System) according to the manufacturer’s instructions, at 1550 psi. MosSCI technique was combined with the bombardment to achieve single copy targeted insertion^24^. Its efficacy is presented in Table S3. The fluorescent tag coding sequences were removed from the constructs to reduce the size of the sequence to be integrated. One week after bombardment, worms with a coordinated movement, suggestive of successful transformation (*unc-119* rescued) were picked and bred in singles. Breeds without uncoordinated progeny over at least three generations were checked by PCR and Sanger sequencing for single copy genomic integration with primers designed for the genomic sequence outside the flanking regions. Those with no integration in the expected MosSCI site, but stable coordinated movements in the offsprings over 5 generations, were considered random integrants and the presence of vector sequence was verified by PCR.

RNA expression was verified by qPCR (LightCycler 480 SYBR Green I Master, Roche) and Sanger sequencing (ABI SeqStudio; *BrilliantDye* Terminator v3.1. Cycle sequencing kit, Nimagen) after total RNA isolation and reverse transcription. Primer sequences available upon request.

### Gentle touch test

The gentle-touch sensation was examined by two researchers (DP, KT), using cat’s whiskers in a blinded fashion^64^. At least 30 animals were tested from each strain, and each animal was touched three times and was considered touch sensitive if reacted to at least two touches. Results were validated in a second experiment by the classic “eyebrow on a toothpick” method in a similarly blinded fashion by two researchers (RL, DP).

### Chemotaxis assay

Chemotaxis assays were performed in two ways: first, as described previously by Margie et al.^33^ and second, by counting the worms in a smaller area around the odorant drop, which reduces the bias of the randomly moving worms near the center (Fig. S3). Four different odorants were tested: diacetyl (1:1000; 12 mM), pyrazine (1mM), butanone (1:1000; 112 mM) and octanol (1:1000; 6.4 mM) the last three of which were the same as in the study of Liang et al.^6^. All odorants were dissolved in ethanol. Control experiments were performed with ethanol in all four quarters. Wild-type (N2) and chemotaxis deficient *odr-3(n2150)* animals were used as negative and positive controls, respectively. A chemotaxis experiment was evaluated if 50–250 worms in the original setup or 20–150 worms in the novel setup were found in all test areas together. At least five conclusive experiments were done for each condition.

‘Smell on a stick’ test was performed based on the method described by Liang et al.^7^.

### Statistical analysis

Gentle touch response and conclusive chemotaxis test rates were compared by Fisher’s exact test. The chemotaxis indexes showed normal distribution as verified by Kolmogorov-Smirnov test. They were compared by one-way ANOVA and Tukey’s Honest Significant Difference post-hoc test.

## Electronic supplementary material

Below is the link to the electronic supplementary material.

Supplementary Material 1

## Data Availability

The mRNA sequences identified during the current study are available in the NLM GenBank database under the following accession numbers: PQ476250 (https://www.ncbi.nlm.nih.gov/nuccore/PQ476250); PQ476249 (https://www.ncbi.nlm.nih.gov/nuccore/PQ476249).
