# Supplementary material for: The MEC-2E isoform with a large C-terminal completely rescues the touch sensation defect of C. elegans
Source: Sci Rep. 2025 Jul 22;15:26606. doi: 10.1038/s41598-025-10711-w (PMC12284166; doi:10.1038/s41598-025-10711-w)
Supplement: Supplementary file 1 — Supplementary Material 1 [file 41598_2025_10711_MOESM1_ESM.docx]

**The MEC-2E isoform with a large C-terminal completely rescues the touch sensation defect of *C. elegans***

Tália Magdolna Keszthelyi^1,2^, Regina Légrádi^1,2^, Dóra Pálya^1,2^, Tímea Köles^1,2^, Ágnes Regős^1^, Dóra Karancsiné Menyhárd^3,4^, Kálmán Tory^1,2, *^

^1^ MTA-SE Lendület Nephrogenetic Research Group, Budapest, Hungary

^2^ Pediatric Center, MTA Center of Excellence, Semmelweis University, Budapest, Hungary

^3^HUN-REN-ELTE Protein Modeling Research Group, ELTE Eötvös Loránd University, Budapest Hungary

^4^Medicinal Chemistry Research Group, HUN-REN Research Centre for Natural Sciences, Budapest, Hungary

[*tory.kalman@semmelweis.hu](mailto:*tory.kalman@semmelweis.hu)

Table S1.

**Nucleotid sequence of codon-optimized *NPHS2* sequence with artificial introns (gray)**

| ATGGAGCGTCGTGCTCGTTCTTCTTCTCGCGAGTCTCGCGGACGCGGAGGACGCACTCCACACAAGGAAAACAAGCGCGCTAAGGCTGAGCGCTCTGGAGGAGGACGCGGACGCCAAGAAGCTGGACCAGAGCCATCTGGATCTGGACGCGCTGGAACTCCAGGAGAGCCACGCGCTCCAGCTGCTACCGTCGTCGATGTTGATGAGGTTCGCGGATCTGGAGAGGAGGGAACTGAGGTTGTCGCCCTTCTTGAGTCTGAGCGCCCAGAGGAGGGAACTAAGTCTTCCGGACTTGGAGCTTGCGAGTGGCTTCTTGTCCTTATCTCCCTTCTTTTCATTATCATGACTTTCCCATTCTCTATTTGGTTCTGCGTTAAGgtaagtttaaacatatatatactaactaaccctgattatttaaattttcagGTTGTTCAAGAGTACGAGCGCGTTATCATCTTCCGCCTTGGACACCTTCTTCCAGGACGCGCTAAAGGACCAGGACTTTTTTTCTTCCTTCCATGCCTTGATACCTACCACAAAGTGGATCTTCGTCTTCAAACTCTTGAAATCCCATTCCACGAAATCGTTACTAAGgtaagtttaaacagttcggtactaactaaccatacatatttaaattttcagGATATGTTCATCATGGAAATCGATGCTATCTGCTACTACCGCATGGAAAACGCCTCCCTTCTTCTTTCCTCCCTTGCTCACGTTTCCAAGGCTGTTCAATTCCTTGTTCAAACCACCATGAAGCGCCTTCTTGCTCACCGTTCTCTTACCGAGATCCTTCTTGAGCGTAAGTCTATCGCTCAAGACGCTAAGGTCGCTCTTGATTCTGTTACTTGCATTTGGGGAATCAAGGTTGAGCGCATCGAGATCAAGGATGTTCGTCTTCCAGCTGGACTTCAACACTCCCTTGCTGTCGAGGCTGAGGCTCAACGTCAAGCTAAGgtaagtttaaacatgattttactaactaactaatctgatttaaattttcagGTTCGTATGATCGCTGCTGAGGCTGAGAAGGCTGCTTCCGAGTCCCTTCGTATGGCTGCTGAGATCCTTTCTGGAACCCCAGCTGCTGTCCAACTTCGTTACCTTCACACCCTTCAATCTCTTTCTACCGAGAAGCCATCTACCGTTGTCCTTCCACTTCCATTCGACCTTCTTAACTGCCTTTCTTCTCCATCTAACCGCACCCAAGGATCTCTTCCATTCCCATCTCCATCTAAGCCAGTTGAACCACTTAATCCAAAGAAGAAGGATTCTCCAATGCTC |
| --- |

**Table S2. Comparison of human podocin and *C. elegans* MEC-2 [1-9] [ensembl.org]**

| **protein** | **human podocin** | ***C. elegans* MEC-2** |
| --- | --- | --- |
| **encoding gene** | *NPHS2* (1q25.2) | *mec-2* (X: [5567754-5590830](https://www.ensembl.org/Caenorhabditis_elegans/Location/View?db=core;g=WBGene00003166;r=X:5567754-5590830)) |
| **isoforms** | single canonical with pathogenic variants in all eight exons | MEC-2A, MEC-2B, MEC-2E  (17 isoforms in ensembl.org) |
| **residues (aa)** | 383 | 481 (MEC-2A)  392 (MEC-2B)  1239 (MEC-2E) |
| **molecular weight (kDa)** | 42.2 | 51.9 (MEC-2A)  42.8 (MEC-2B)  136.5 (MEC-2E) |
| **expressing cell type** | podocyte | six mechanosensory neurons (MEC-2A, MEC-2E), olfactory neurons (MEC-2B) |
| **subcellular localization** | plasma membrane | plasma membrane |
| **structure** | one intramembrane region (105-121. aa), intracellular N- and C-terminal;  SPFH domain (122-344);  262 aa long C-terminal tail with 3 helical regions | one intramembrane region (114-136. aa);  intracellular N- and C-terminal;  SPFH domain (160-361);  345 (A); 256 (B); 1103 (E) aa long C-terminal tail |
| **molecular associations** | oligomerization; part of a multiprotein complex (nephrin, CD2AP, TRPC6) | oligomerization; part of a multiprotein complex (MEC-4, MEC-6, MEC-10) |
| **presumptive function** | organisation of the multiprotein complex in lipid rafts of the plasma membrane, cholesterol binding, ion channel regulation (TRPC6) | organisation of the multiprotein complex in lipid rafts of the plasma membrane, cholesterol binding, ion channel regulation (MEC-4, MEC-10) |
| **related phenotype** | steroid-resistant nephrotic syndrome | mechanosensation defect |
| **interallelic interactions** | [4, 5, 10] | [11] |

**Table S3. Efficiency of the MosSCI technique in genomic integration when combined with microparticle bombardment**

| encoded isoform | bombardment events (n) | worms with a rescued, coordinated motion (n) | MosSCI integrant strains (n) | random integrant strains (n) |
| --- | --- | --- | --- | --- |
| MEC-2A | 5 | 27 | 1 | no data^#^ |
| MEC-2E | 5 | 11 | 0 | 3 |
| MEC-2E with premature stop | 2 | 10 | 0 | 4 |
| NPHS2([NM_014625.4](http://www.ncbi.nlm.nih.gov/nuccore/NM_014625.4)) | 4 | 13 | 1 | no data^#^ |
| ‘genomic giant vector’ | 20 | 28 | 0 | 0 |

^#^ Random integrant strains were not searched any more once a MosSCI integrant strain was successfully generated

**Figure S1. PCR reactions for checking the isoforms listed in ensembl.org**

**
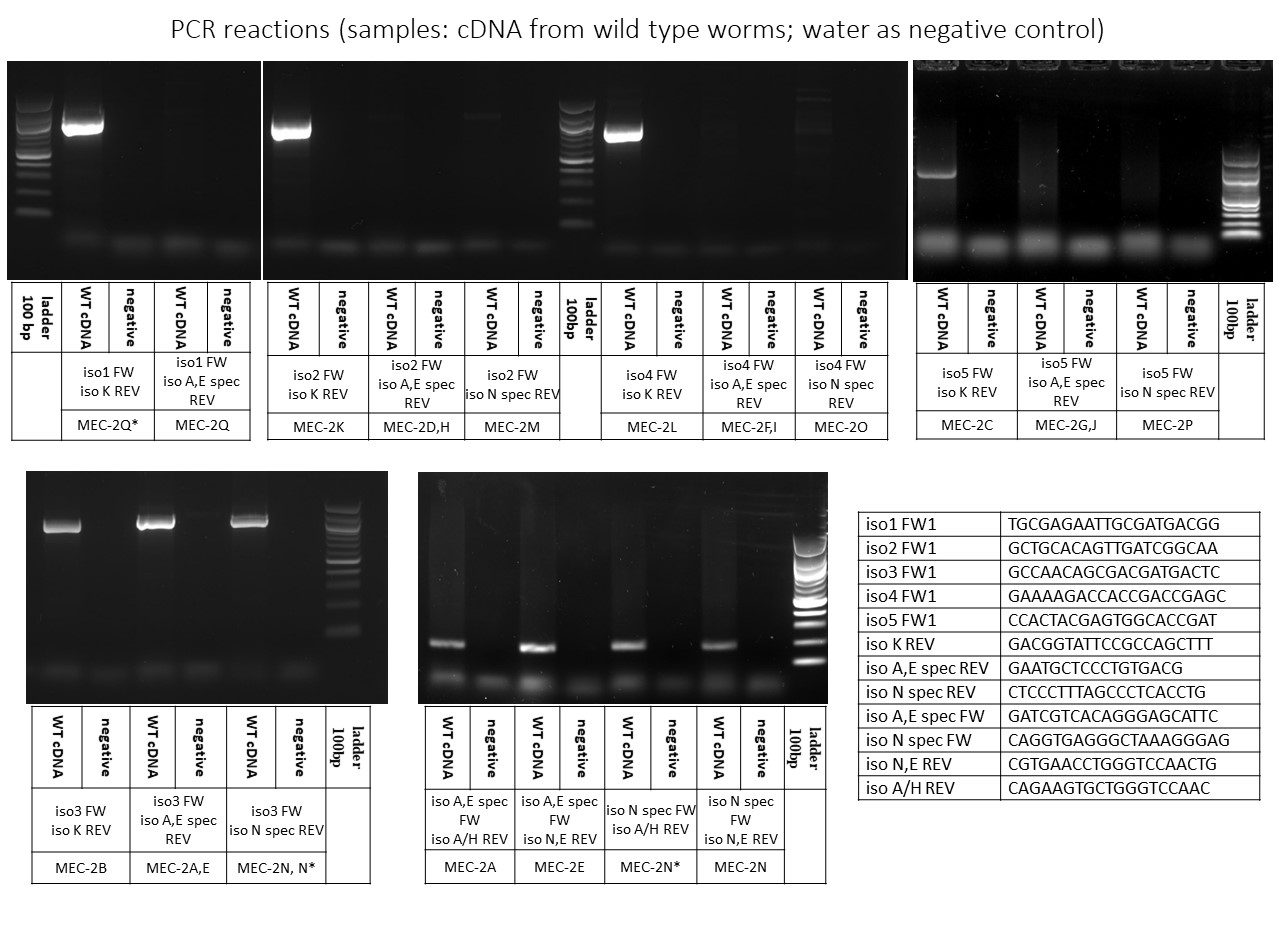
**

**Figure S2.** A. **Predicted structure of MEC-2E** (115-1239 segment shown). Surface was drawn for the PHB domain and H1 and H2 helices for which the accuracy of the model is highest. Pro1026 – the truncation site – is shown explicitly, the 1027-1239 segment is colored deep blue –lilac-magenta. B. Predicted structure of the MEC-2E tetramer. The proximity of the chains enhanced helicalization – the 1027-1239 segment is shown to form a helical “buttress” supporting the aligned PHB domains.


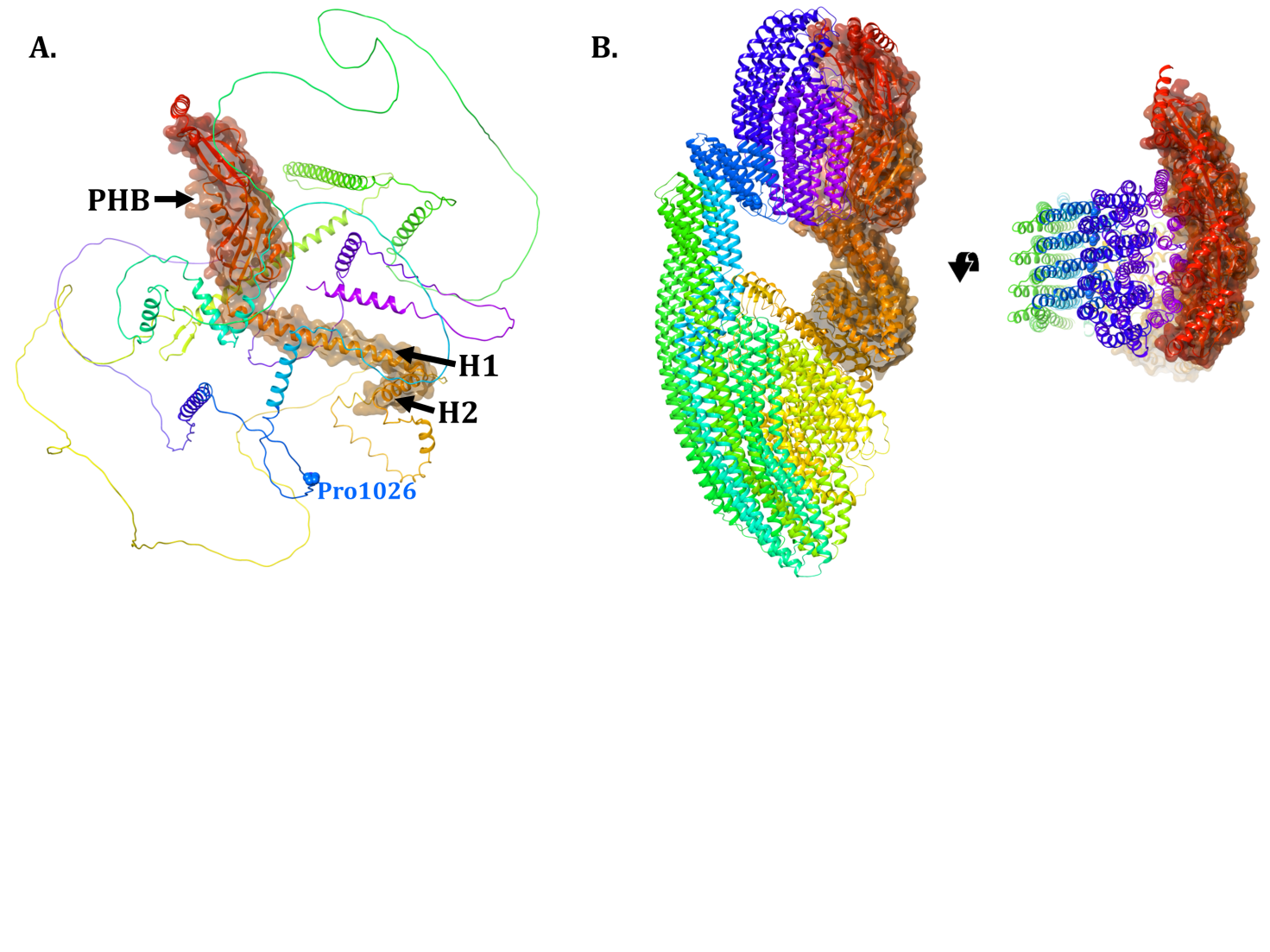


**Figure S3. Comparison of chemotaxis assays**


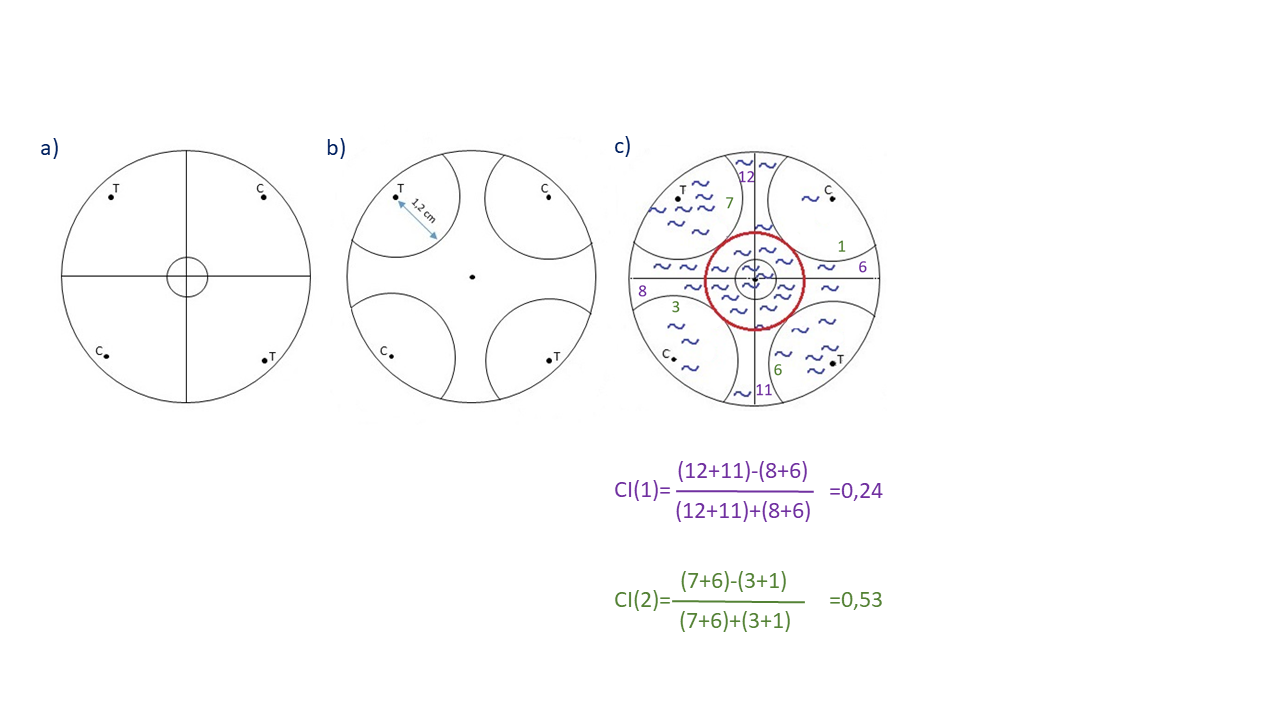


The circles represent petri dishes with a diameter of 60 mm. ‘T’= test-odorant, ‘C’= control-odorant.

a) According to the method of Margie et. al.[12], worms are placed to the centre, and 30 minutes later the worms are counted in the four quarters outside of the central circle.

b) Proposed method herein: worms are placed to the centre, 30 minutes later the worms are counted in the four ‘semicircles’ around the odorants.

c) Illustration of the difference between the two methods demonstrating the biasing effect of centrally localized, randomly moving animals (violet numbers: counted worms in the quarters; green numbers: counted worms in the semicircles)

1. Huber TB, Schermer B, Benzing T: **Podocin organizes ion channel-lipid supercomplexes: implications for mechanosensation at the slit diaphragm**. *Nephron Exp Nephrol* 2007, **106**(2):e27-31.

2. Huber TB, Schermer B, Muller RU, Hohne M, Bartram M, Calixto A, Hagmann H, Reinhardt C, Koos F, Kunzelmann K *et al*: **Podocin and MEC-2 bind cholesterol to regulate the activity of associated ion channels**. *Proc Natl Acad Sci U S A* 2006, **103**(46):17079-17086.

3. Völker LA, Schurek EM, Rinschen MM, Tax J, Schutte BA, Lamkemeyer T, Ungrue D, Schermer B, Benzing T, Höhne M: **Characterization of a short isoform of the kidney protein podocin in human kidney**. *BMC Nephrol* 2013, **14**:102.

4. Straner P, Balogh E, Schay G, Arrondel C, Miko A, L'Aune G, Benmerah A, Perczel A, D KM, Antignac C *et al*: **C-terminal oligomerization of podocin mediates interallelic interactions**. *Biochim Biophys Acta Mol Basis Dis* 2018, **1864**(7):2448-2457.

5. Tory K, Menyhard DK, Woerner S, Nevo F, Gribouval O, Kerti A, Straner P, Arrondel C, Huynh Cong E, Tulassay T *et al*: **Mutation-dependent recessive inheritance of NPHS2-associated steroid-resistant nephrotic syndrome**. *Nat Genet* 2014, **46**(3):299-304.

6. Liang X, Calovich-Benne C, Norris A: **Sensory neuron transcriptomes reveal complex neuron-specific function and regulation of mec-2/Stomatin splicing**. *Nucleic Acids Res* 2022, **50**(5):2401-2416.

7. Nakano S, Ikeda M, Tsukada Y, Fei X, Suzuki T, Niino Y, Ahluwalia R, Sano A, Kondo R, Ihara K *et al*: **Presynaptic MAST kinase controls opposing postsynaptic responses to convey stimulus valence in Caenorhabditis elegans**. *Proc Natl Acad Sci U S A* 2020, **117**(3):1638-1647.

8. Nakano S, Nakayama A, Kuroyanagi H, Yamashiro R, Tsukada Y, Mori I: **Genetic screens identified dual roles of MAST kinase and CREB within a single thermosensory neuron in the regulation of <em>C. elegans</em> thermotaxis behavior**. *bioRxiv* 2022:2022.2007.2012.499830.

9. Calixto A, Ma C, Chalfie M: **Conditional gene expression and RNAi using MEC-8-dependent splicing in C. elegans**. *Nat Methods* 2010, **7**(5):407-411.

10. Miko A, D KM, Kaposi A, Antignac C, Tory K: **The mutation-dependent pathogenicity of NPHS2 p.R229Q: A guide for clinical assessment**. *Hum Mutat* 2018, **39**(12):1854-1860.

11. Chalfie M, Sulston J: **Developmental genetics of the mechanosensory neurons of Caenorhabditis elegans**. *Dev Biol* 1981, **82**(2):358-370.

12. Margie O, Palmer C, Chin-Sang I: **C. elegans chemotaxis assay**. *J Vis Exp* 2013(74):e50069.
